# Standardising Access to Sensitive Data with Machine-Actionable Access Conditions

**DOI:** 10.23889/ijpds.v11i1.3454

**Published:** 2026-09-17

**Authors:** Lucas van der Meer, Ahmad Hesam, Jetze Touber, Ricarda Braukmann, Deborah Thorpe, Emilie Kraaikamp, Tom Emery

**Affiliations:** 1 Erasmus University Rotterdam, Burg. Oudlaan 50, 3062 PA, Rotterdam, the Netherlands; 2 SURF, Moreelsepark 48, 3511 EP, Utrecht, the Netherlands; 3 DANS, Anna van Saksenlaan 51, 2593 HW, Den Haag, the Netherlands; † These authors contributed equally to this work

**Keywords:** machine-actionable access conditions, open digital rights language, Trusted Research Environment, data access broker

## Abstract

**Introduction:**

While many countries have invested heavily in secure technical infrastructures such as Trusted Research Environments (TREs), the rules that determine who may access which data, under what conditions, and for which purposes are still expressed primarily as natural language legal and administrative documents, with little or no accompanying machine-readable representation. This limits automation, interoperability, and scalability, particularly for research that spans multiple data holders.

**Objectives:**

This paper examines these challenges through the Dutch social science data landscape and presents a framework for standardising access governance using machine-actionable access conditions, with the aim of bridging the gap between secure technical environments and scalable access governance.

**Methods:**

Building on the Open Digital Rights Language (ODRL), we propose a library of reusable access condition templates and a Data Access Broker service. The framework was developed through iterative consultation with data holders in the Dutch SSH domain and draws on ongoing developments within the European Open Science Cloud (EOSC) and related data sharing initiatives.

**Results:**

We demonstrate that machine-actionable access conditions can operationalise legal and organisational oversight in a transparent, auditable, and interoperable way without replacing it. The proposed framework addresses key challenges identified in the Dutch context — including fragmentation, administrative burden, and inconsistent decision-making — and is applicable across European data infrastructures.

**Conclusions:**

The approach does not replace legal or organisational oversight, but operationalises it in a transparent, auditable, and interoperable way. While grounded in the Dutch context and the ODISSEI infrastructure, the challenges addressed are common across Europe. The paper presents a concrete implementation pathway supporting ongoing developments within EOSC and related data sharing initiatives.

## Highlights

- Many European countries have invested in Trusted Research Environments, yet access governance remains largely manual, fragmented, and expressed in natural language.
- Using the Dutch social science data landscape as a case, we identify core challenges: fragmentation, administrative burden, inconsistent conditions, and data holder concerns.
- We propose a framework of machine-actionable access conditions based on ODRL, comprising reusable access templates and a Data Access Broker service.
- The framework operationalises existing governance transparently and interoperably without replacing legal or organisational oversight.
- While grounded in the Dutch ODISSEI infrastructure, the approach is applicable across Europe and aligns with EOSC and EHDS developments.

## Introduction

Access to detailed population and social science data is essential for understanding demographic change, social inequality, health trajectories, and the effects of public policy. An increasing share of this research relies on sensitive microdata from administrative registers, surveys, and linked sources [1, 2]. Many datasets are sensitive because they contain privacy-sensitive information, are subject to copyright, or hold economic value to the data holder [3].

Although these data offer substantial analytical value, they are subject to legal, ethical, and organisational constraints that require careful data governance. As a result, access is typically mediated through restrictive procedures intended to balance scientific utility with the protection of individuals, organisations, and intellectual property [4].

In practice, current data access arrangements often fail to achieve this balance efficiently or transparently. Researchers can encounter fragmented procedures, inconsistent interpretations of legal requirements, and long approval timelines, particularly when projects involve multiple data holders. These barriers are well documented in the social science literature and persist even in countries with advanced statistical systems and secure research infrastructures.

Over the past decade, substantial investments have been made in technical infrastructures to mitigate disclosure risks. Trusted Research Environments (TREs), remote secure analysis facilities, and controlled output checking are now standard in many countries [5]. While these infrastructures effectively address technical risks, procedural and governance challenges remain. Although TRE operators have developed mature governance processes around access review, researcher accreditation, and disclosure control, these processes often remain organisation-specific, manual, and difficult to coordinate across multiple data holders. Decisions about who may access which data, under what conditions, and for which purposes remain largely manual, arbitrary, bespoke, and reliant on human interpretation.

One reason for this fragmentation is that access conditions are expressed primarily as natural language legal and administrative documents, with little or no machine-readable representation. Licences, access policies, and user agreements are written as legal or administrative text intended for committees and data stewards. Although legally necessary and crucial for human understanding of the metadata, such documents are poorly suited to automation, interoperability, or systematic comparison. As a result, access workflows depend on ad-hoc communication and case-by-case judgement, limiting scalability, increasing turnaround times, and reducing transparency.

In this paper, we analyse the current European landscape of Trusted Research Environments and show that, despite substantial investments in secure technical infrastructures, the procedures that govern access to sensitive data remain largely manual, fragmented, and expressed in natural language. Using the Dutch research data ecosystem as a concrete case, we identify the resulting challenges for researchers and data holders, including delays, administrative burden, and inconsistent decision-making. We then propose a technical and governance framework for standardising data access through machine-actionable access conditions based on Open Digital Rights Language (ODRL)^1^, comprising a library of reusable access templates and a Data Access Broker service that interprets these conditions and orchestrates access workflows across repositories and secure environments. Rather than replacing legal or organisational oversight, this approach operationalises existing governance in a transparent, auditable, and interoperable manner. The paper concludes by outlining how this access-focused framework can be extended in future work to address use conditions and policy alignment, and by situating the proposal within broader European efforts to develop scalable standards for sensitive data access.

## The Current European Landscape

Across Europe, efforts to balance protection of sensitive data with research access are increasingly being shaped by developments within the European Open Science Cloud (EOSC). While national systems vary widely, EOSC has become the primary arena in which shared solutions for interoperability, transparency, and automation of access conditions are being articulated [6]. Despite significant progress, most countries still rely on human-readable licences and manual approvals, highlighting a persistent gap between secure technical infrastructures and machine-actionable governance.

Several EOSC initiatives explicitly address this gap. EOSC Beyond is developing a Digital Rights Vocabulary and the OpenREL toolkit to represent access conditions and reuse conditions, alongside an execution framework that allows services to act on such rights programmatically [7]. In parallel, EOSC EDEN focuses on automated curation and long-term preservation, embedding licence selection and policy enforcement into repository workflows and aligning practices across the EOSC federation [8]. Together, these initiatives provide the semantic and technical foundations for expressing access conditions in ways that can be interpreted consistently across infrastructures. However, they remain largely infrastructural and generic: translating these capabilities into operational access workflows for sensitive population data still depends on a national implementation.

Against this EOSC backdrop, European countries have adopted different governance models for sensitive data access, ranging from highly centralised authorities to federated systems coordinated through shared frameworks. In 2019, Finland enacted dedicated legislation for the secondary use of health and social data, establishing the Findata Data Permit Authority [9]. Findata functions as a single-entry point for researchers requesting data from multiple registries, coordinating access across hospitals, registries, and other data holders. This model has significantly reduced fragmentation and delays, with waiting times for multi-source data access falling from up to fourteen months to a few months on average. Access conditions are standardised through law and permit requirements, and data are delivered through secure processing environments operated by the national IT centre (CSC). While Findata’s permits are not expressed in a formal machine-readable policy language, the centralised mediation ensures consistency. All researchers operate under a common, legally defined set of conditions.

The United Kingdom represents a contrasting, federated model that nonetheless aligns closely with EOSC principles of distributed governance and interoperability. For administrative and government-held data, rather than a single access authority, the UK relies on shared frameworks such as the Five Safes and standardised access tiers (Open, Safeguarded, and Controlled) used across organisations including the UK Data Service and the Office for National Statistics (ONS). These tiers clarify broad expectations around data sensitivity, researcher accreditation, and security requirements, with Controlled data requiring approved projects and analysis within secure environments. However, beyond these high-level labels, access conditions remain largely bespoke to individual repositories, and approval processes are predominantly manual, such as through the ONS Research Accreditation Service under the Digital Economy Act. Health data follows separate legal and governance arrangements and remains considerably more fragmented across organisations and TREs. At the same time, the UK is among the most active in exploring machine-actionable approaches: the UK Data Service is piloting the use of ODRL to formally encode access conditions, explicitly positioning W3C standards to improve interoperability across repositories [10].

France has similarly pursued alignment through centralisation, particularly for health data, via the Health Data Hub (HDH) [11]. The HDH is intended as a single platform through which researchers can request access to a wide range of national health datasets, hosted within a common secure infrastructure. While its implementation has faced legal and organisational challenges, the model reflects a strong commitment to standardised workflows, unified metadata catalogues, and central evaluation of requests. As in Finland, access conditions are primarily defined through legislation, reducing the need for negotiation with individual data holders. Although not framed in machine-readable policy terms, the HDH illustrates a broader European shift toward one-stop access under common rules – a direction strongly reinforced by EOSC and European Health Data Space (EHDS) ambitions.

Many other European countries fall between these centralised and federated extremes. Sweden, Denmark, and Norway all operate rich national registries and robust secure analysis environments, often aligned with Five Safes principles. However, access governance remains largely domain-specific, with separate authorities responsible for health, social, and economic data. While procedures are efficient within domains, cross-domain linkage remains difficult in the absence of a shared access framework or interoperable condition model.

In parallel to these national developments, the European Commission is advancing the European Data Spaces framework, including initiatives such as SIMPL to enable secure and interoperable data exchange across sectors. These efforts focus primarily on infrastructure for identity, access control, and policy enforcement between organisations. While highly relevant, they do not yet address the detailed, standardised representation of research-specific access conditions and workflows. Machine-actionable access conditions for research data can therefore be seen as a complementary layer, translating domain-specific governance requirements into structured policies that could interoperate with broader European Data Spaces infrastructures.

The first major operationalisation of European Data Spaces is in the health domain through the proposed EHDS [12]. Findings from the TEHDAS joint action describe a heterogeneous landscape: access procedures vary widely, fewer than half of countries have national data catalogues, and no member state is fully prepared for EHDS implementation [13]. While most countries have invested in secure processing environments, far fewer have standardised access conditions or established single access bodies. EHDS is expected to require each country to designate a Health Data Access Body and apply common rules across data holders, reinforcing the need for structured, interoperable representations of access conditions – precisely the gap EOSC initiatives aim to fill.

Beyond Europe, similar developments reinforce this trajectory. The Global Alliance for Genomics and Health has developed the Data Use Ontology (DUO) to standardise permitted uses of genomic data, with active work mapping these terms into formal policy languages [14]. In the industrial domain, the International Data Spaces framework uses ODRL-based contracts to automate enforcement of data sharing constraints [15]. These efforts demonstrate that machine-actionable access governance is technically feasible and increasingly expected across domains.

Taken together, EOSC provides the unifying layer through which these diverse national approaches can converge. While countries differ in governance structure, they face a common challenge: secure infrastructures alone do not resolve fragmentation, opacity, and administrative burden in access decision-making. Machine-actionable representations of access conditions, developed within EOSC and implemented nationally, offer a credible path toward scalable, transparent, and interoperable access to sensitive research data.

Against this background, this paper presents a solution for implementing machine-actionable access conditions within the Dutch social science infrastructure. It combines formal policy languages with an intermediary service that interprets access conditions and orchestrates workflows across data repositories and secure environments. While grounded in the Dutch context and ODISSEI^2^, the challenges addressed are common internationally, and the solution is intended to inform broader efforts to modernise access governance.

## Challenges in the Dutch Sensitive Data Landscape

The Netherlands has a mature data landscape, yet access to these data is governed through a highly decentralised system. Unlike more centralised models such as those in Finland or France, the Netherlands has no single national access authority or unified permit regime for sensitive research data. Instead, governance is distributed across many independent data holders, each operating under its own legal interpretations, access policies, review procedures, and contractual arrangements. While this reflects strong institutional autonomy, it also results in a fragmented access landscape. In practice, researchers must navigate multiple, often uncoordinated access routes. Statistical microdata, health registries, cohort studies, and domain-specific collections each follow distinct procedures, and cross-domain linkage typically requires additional intermediaries and approvals. Even where datasets are discoverable through national portals like the ODISSEI Portal^3^, access decisions remain organisation-specific and largely manual.

This fragmentation contrasts sharply with the Netherlands’ substantial investment in secure technical infrastructures. Trusted Research Environments, remote access facilities, and output checking are well established and widely trusted. However, the rules that govern who may access which data, under what conditions, remain largely expressed in natural language and bespoke, limiting automation, reuse, and interoperability. The Dutch case therefore highlights a core governance gap of mature technical safeguards without an equally standardised, scalable access layer. Machine-actionable access conditions offer a way to formalise and operationalise access rules across institutions, enabling consistency, transparency, and more efficient use of secure infrastructures without undermining existing legal responsibilities or institutional autonomy. In this section we summarise the challenges faced in the Netherlands as a use case for implementing machine-actionable access conditions.

### Fragmentation of Data Governance and Access Procedures

The Dutch research data ecosystem is rich in data but highly fragmented in terms of governance and access procedures. Health data, for example, a common type of sensitive data, are collected by many entities – Statistics Netherlands maintains statistical microdata and health-related registries [16]; RIVM holds epidemiological and survey datasets; over 100 separate national healthcare quality registries each manage their own data; and biobanks are decentralised across hospitals. There is no centralised catalogue or unified portal that provides an overview of all these data collections or how to request access to them. Researchers must approach each data holder individually, navigating different forms and criteria for each. Data in social sciences and humanities are typically available in centralised catalogues and portals such as the ODISSEI Portal which provide an overview across various data holders but also have not yet unified the access requests to the data. According to a recent European assessment, the typical data access procedure involves first obtaining necessary ethical approvals, then writing a research protocol, and finally applying to each relevant data holder separately for the needed datasets. This siloed approach means duplicated effort and uncertainty, especially for studies that require combining data from multiple sources.

### Inconsistent, Complex, and Arbitrary Conditions

Because there is no standard data access conditions framework, each data holder sets its own terms and interpretations of data protection rules. Although many data holders may have similar access conditions, they all implement them in a different way and therefore have different access procedures. The legal basis for using personal data in research is often interpreted differently across institutions, leading to “continuous discussions and different procedures” in each case [13]. For instance, Dutch law (AVG, the national implementation of GDPR) allows certain research uses without explicit consent under strict conditions, but in practice those conditions are understood variably by different organisations. An example of this is the analysis of microdata via Statistics Netherlands under the ‘CBS Wet’^4^.

Some data holders might require written consent or an extensive data use agreement even when not required by law, while others might rely on an opt-out system. Differences in how organisations interpret the legal basis for data sharing result in inconsistent access conditions and a fragmented landscape of restrictions. This inconsistency makes it hard for data consumers to predict what will be required for access, and for holders to know if their safeguards align with national best practices. It also makes it difficult for data consumers to search for datasets with access conditions that meet their needs, for instance, one in which they can visually inspect the data. Besides, it is common that datasets have no publicly available documentation on access conditions at all, which results in arbitrariness of who is granted access (see also analysis by [17]).

### Lengthy Delays and Administrative Burden

The fragmented landscape directly translates into slow and cumbersome access, or no access at all. Data holders want to spend as little time as possible on data access requests. Researchers often face long turnaround times for data requests and it is not uncommon for approvals to take many months. Each data source may have its own access committee or review board, leading to sequential or parallel approval processes that significantly delay projects. Moreover, fees and costs differ between data holders, and each may charge separately for data preparation or access.

For example, Statistics Netherlands requires an institution to be accredited and charges for the use of its secure microdata environment. Some health registries charge data extraction fees while others require data consumers to arrange a trusted third party for pseudonymisation at the data consumer’s expense. There is little harmonisation and a recent country analysis noted that “time and fees for accessing data vary between data holders” [13], even for similar types of requests. Findings from a recent analysis of Dutch access procedures for restricted access data similarly show that researchers often face long and unpredictable turnaround times, repeated information requests, and administrative processes that differ substantially between organisations [17]. Related work on perceived barriers to access highlights that unclear steps, inconsistent communication, and prolonged waiting times can discourage researchers, particularly those early in their careers, from pursuing data-intensive projects [18]. This variability makes access inherently fragile. The more time and coordination required, the higher the chance that access is delayed or not completed at all. This applies especially to data holders for whom providing scientific access to data is not part of their core business.

### Data Holder Concerns and Control

On the side of data holders, a key challenge has been ensuring that once data is shared, it remains protected. Understandably, organisations like the Dutch National Institute for Public Health and the Environment (RIVM) or Statistics Netherlands are cautious about releasing sensitive data, fearing misuse or identification of individuals. Historically this led to restrictive access modes. For instance, Statistics Netherlands only allows analysis of microdata within a secure remote environment and reviews all output before release. Many health data holders insist on project-specific contracts that tightly circumscribe usage. Holders have lacked a common technical solution to confidently share data while still retaining control. This is where infrastructure like SURF’s Secure ANalysis Environment (SANE)^5^ has started to help. SURF is the national computing centre in the Netherlands that developed SANE, a Trusted Research Environment (TRE) where data can be analysed without ever leaving the controlled server, giving holders confidence that data consumers cannot leak or misuse the raw data. SANE addresses the challenge of data holders hesitating to share sensitive datasets with researchers due to concerns about losing control over the data by allowing holders to retain full control. However, while SANE and similar TREs mitigate the technical risk of data sharing, the access condition fragmentation remains: each holder separately decides if and how data gets into such an environment in the first place. Without a standardised access condition, a data consumer might get one dataset into SANE relatively easily while another dataset’s holder draws the process for months. In short, the tools for secure analysis are emerging, but a unified approach to granting access under consistent conditions is missing.

### Complexities in Data Linkage and Reuse

Because each dataset comes with distinct conditions, combining data for richer analysis is particularly challenging. Linking person-level data (i.e. that which contains information about specific individuals) across sources often requires using the citizen service number (BSN) via a trusted third party for privacy reasons. The logistics of arranging such linkages multiply the administrative steps. Data consumers might need separate approvals from each data source to export identifiers to the trusted party and then re-import the linked data into a secure environment. In some cases, data cannot be easily shared across domains at all due to incompatible access conditions or a lack of cross-recognition of their agreements. Thus, valuable research questions that span multiple datasets are hindered by the decentralised management of Dutch data and the absence of an integrated access condition framework.

These challenges all point to a need for a more streamlined and uniform system. In the current model, significant human effort is spent interpreting and negotiating terms for each data request.

## Standardising Data Access Conditions with a Machine-Actionable Model

For a number of years, ODISSEI, SURF and DANS have been working with a large number of organisations that hold sensitive data to determine which challenges they face and what would be the right access conditions. The consultation process included four workshops (November 2022, June 2023, November 2024 and February 2025) in which key data holders from the SSH domain were represented. Some data holders were willing to make sensitive data available for research purposes but did not know how and wanted to spend as little time as possible on the issue. Others were sharing sensitive data more structurally but were interested in making their process more efficient. The data holders’ input formed the basis for the challenges and proposed solutions as outlined here. ODISSEI, DANS, and SURF developed a first version of a Data Access Broker^6^ in conjunction with the published access template for TRE-only usage of a sensitive dataset.

To address the challenges described above and to build on the emerging European momentum, a technical framework for standardising data access conditions using ODRL is adopted. Our approach focuses on the *machine-actionable* representation of access rules and workflows, which can substantially reduce fragmentation and administrative effort. It does not replace the need for clear governance, policy alignment, nor is it a one-stop-shop authority. Instead, it provides the infrastructure layer that such governance models can build upon. By expressing permissions, prohibitions, and obligations in a common digital format, and linking them to automated access and curation services, this framework enables consistent, transparent, and auditable data access processes across institutions.

ODRL offers a widely recognised standard for defining policies governing the use of digital content, making it a suitable basis for encoding data access conditions. Within ODRL, policies consist of *permissions* (allowed actions), *prohibitions* (forbidden actions), and *obligations* (required actions), which can be extended with *constraints* such as time limits, purposes, or jurisdictions. Access management only deals with a subset of the policies’ conditions: those that must be fulfilled to obtain access. By formalising these conditions in machine-readable form, ODRL enables technical systems such as data portals or broker services to automatically verify compliance and initiate access under the agreed conditions. From a technical standpoint, ODRL conditions are interoperable and extensible. ODRL is a W3C recommendation with semantic richness, meaning these standard conditions can be shared across systems and internationally. A well-crafted ODRL condition is machine-interpretable and thus machine-actionable, fulfilling FAIR principles for interoperability [19].

This section outlines how ODRL can be applied in practice through two interlinked components. First, we propose Ready-to-use Access Conditions composed of fixed sets of ODRL rules, being a library of standard data access conditions expressed in a formal language and endorsed in our community. These conditions can be taken as a use case for further standardisation in the context of the EOSC, particularly to standardise rights vocabularies. Second, a Data Access Broker service is an intermediary platform that interprets these ODRL conditions and automates the access request workflow, including delivering data to secure research environments when conditions are met. Together, these components aim to transform the currently manual, case-by-case negotiation of data access into an automated, transparent, and enforceable process. We detail the two components below.

While the technical components described here are necessary to enable machine-actionable access governance, they are not sufficient on their own to ensure adoption. Widespread uptake will require sustained coordination, explanation, and alignment with the policies and practices of major data holders. This includes engagement with legal teams, access committees, and governance bodies to build shared understanding and trust in machine-actionable access conditions. National research infrastructures such as ODISSEI and CLARIAH are well positioned to take on this role, acting as conveners between data holders, repositories, and service providers. By coordinating governance discussions, developing shared guidance, and iteratively refining access templates in collaboration with stakeholders, these infrastructures can help translate the technical solution into an accepted and operational standard.

### Ready-to-use Access Condition Templates (Standard ODRL Rule Sets)

The first part of the solution is to develop a set of standard access condition templates for research data usage, each composed of a well-defined combination of ODRL rules. By adopting these standard access templates, data holders benefit from consistency and legal clarity.

The idea is to distil the common scenarios of data sharing into a handful of reusable access conditions. Data holders could then choose the access conditions that match their dataset, rather than drafting terms from scratch or improvising conditions each time. This is analogous to how Creative Commons licences standardised the sharing of creative works – here we aim to standardise the sharing of sensitive *research datasets* under consistent conditions.

The key is that each access template is composed of a common library of access conditions. In practice, each access condition would be an ODRL policy that encodes the condition. The following combinations of access conditions are used frequently by data holders that make their data available for scientific purposes [20]:

- A **“Public Use with Registration”** access template is a permissive template for less sensitive datasets (for example, partially anonymised or aggregated data). It allows downloading the data to the data consumer’s own system (i.e. a permission to copy), as long as the consumer meets the obligations to verify it is a researcher and to register at the controller. These conditions are used a lot across the scientific domain, for instance with the European Social Survey and the LISS panel.
- A **“TRE-only”** access template could permit a data consumer to see and analyse the data for scientific research purposes but prohibits downloading the data to their own unsupervised environment. This is under the obligations that the consumer has an affiliation with a research institution, and that it accepts the provider’s terms and conditions. These conditions are used frequently by National Statistical Institutes and are often summarised as the Five Safes principles. This framework can then be enforced by allowing analysis only within an *approved TRE* like SANE.
- A **“Blind Analysis”** access template imposes stricter access conditions. It generally prohibits *direct viewing of data* by the data consumer. Obligations are that data consumers have completed specific training or have certain clearance and are qualified users. These conditions are commonly used in the health sciences or accessing collections whose distribution and access is restricted under intellectual property law.

In addition, access templates can be stacked on top of one another. For instance, data holders might add to the TRE-only template that they obligate a research proposal, or that they only allow non-commercial use.

For most data repositories, there is a finite number of building blocks for the majority of access-related activities. By identifying those core building blocks (e.g., actions like *use, read, copy, aggregate*), we can mix-and-match them into templates covering most scenarios. This approach is being pursued by the UK Data Service, which is establishing core ODRL vocabularies for repository access levels. Similarly, EOSC Beyond is standardising access conditions by defining rights vocabularies [7]. The proposed solution here aligns with and builds on these efforts by defining Dutch standard rule sets once and then making them available for reuse. Having a finite number of access conditions will also allow data consumers to filter for datasets with a specific access condition, for instance one where the data consumer can tinker with the data.

It is important to emphasise that these three examples are not intended to be exhaustive. They illustrate common patterns, but they do not limit what can be expressed in ODRL, nor what access templates data holders may wish to adopt. Repositories may draft additional templates that reflect their own domain practices or policy requirements, and depositors can select from those templates when publishing their data. The aim is therefore not to prescribe a closed set of access templates, but to provide a shared vocabulary and structure so that any template, standard or custom, can be expressed consistently and interpreted automatically.

Each standard access condition has a human-readable counterpart (a plain language description) so that data consumers and data stewards can easily understand it. For instance, an access condition might be titled “Secure Analysis – Research Only – Non-Commercial” with the description “Researchers may analyse this dataset for non-commercial research within an approved secure environment. No personal data may leave the environment; outputs will be checked by the data holder. The data consumer must sign a confidentiality agreement.” The *exact same terms* would be encoded in the ODRL condition file attached to the dataset’s metadata. This ensures transparency – humans and machines see the same rules, just expressed in different formats.

Instead of reinventing conditions for each data request, an organisation willing to make data available could pick the access condition that suits their dataset’s sensitivity, ensuring consistency and legal clarity. If needed, a holder can still have multiple tiers of data access by using different templates for different versions of data (e.g., a more detailed dataset under a stricter template, a de-identified summary under a lighter one). The availability of templates also makes it easier to get organisational buy-in: once the standard access conditions are vetted by legal and ethical boards, data stewards don’t have to seek fresh approval each time – using a standard condition becomes routine.

Uniform access templates could also result in more datasets being available to researchers. Seeing and being able to easily adopt the same access templates of a variety of data holders can give other data holders the confidence to also make currently closed data available.

The creation and publication of ready-to-use access templates are adopted by domain research infrastructures, such as ODISSEI (social sciences in the Netherlands) and CLARIAH (humanities in the Netherlands). In the health domain, a future Health Data Access Body (HDAB) could also take on this coordinating role. By formally endorsing or maintaining standardised access templates, such as ODISSEI’s Five Safes licence [21], the research infrastructures help ensure that holders apply consistent conditions, reducing interpretation differences and lowering administrative burdens for both holders and applicants.

In summary, ready-to-use ODRL-based access templates can contribute to greater uniformity. They turn the unclarity of access conditions into explicit rule sets that can be commonly understood. They significantly reduce fragmentation of access conditions by offering a small set of standard, reusable conditions.

### Data Access Broker service

A crucial pillar of the solution is the Data Access Broker – a service that mediates between data holders and data requesters by interpreting the data access conditions and managing the corresponding workflows. If the standardised access conditions and ODRL conditions are the rules of the game, the Broker is the referee and facilitator that applies those rules in practice. The Data Access Broker is an online service where data consumers submit data access requests and data holders review these requests and respond to them. The Broker has several key functions:

- **Condition Matching:** When a data consumer submits an access request for a dataset (or multiple datasets), the Broker retrieves the ODRL access template from the dataset’s metadata, which contains the applicable access conditions. For this mechanism to function, these conditions must be embedded in the dataset metadata. This requires data holders to publish their metadata in a trustworthy repository that supports the expression of conditions in ODRL. The Broker then validates the submitted request details against the access conditions specified by the data holder. If the access conditions include obligations (e.g., “data consumer must be affiliated with a Dutch university” or “must have ethics approval”), the Broker verifies these by retrieving relevant information from external information systems, such as federated identity and access management infrastructures used by research institutions. Because the access templates outlined in the previous section are machine-readable, the Broker can parse them automatically and perform an initial automated triage. If a request clearly violates one or more access conditions, the Broker can immediately deny the request or prompt the applicant to revise it. Conversely, if all conditions are satisfied (i.e., the user meets the constraints and accepts all obligations), the Broker can advance the process by issuing a positive recommendation to the data holder. This form of machine-to-machine comparison aligns with the FAIR Data Train concept, in which “the data station has access conditions described in a Rights Expression Language […] and the train’s access request is compared to check whether they match” [22]. The Broker operationalises this matching process in a standardised manner across all datasets.
- **Workflow Automation:** Once a request is approved – either automatically because it satisfies all access conditions, or after manual approval by the data holder where human oversight is required – the Broker orchestrates the data access workflow by invoking APIs of the relevant infrastructure services. For datasets with *public-use-after-registration* access conditions, the Broker initiates the transfer of the dataset from the data holder’s storage system to the consumer’s designated computing environment (e.g., a local workstation or cloud environment). For datasets restricted to Trusted Research Environment (TRE) access, the Broker coordinates the controlled ingestion of the dataset into the TRE. Beyond data transfer, the Broker can request configuration of the compute environment with the required software and tools. Structured metadata technologies such as RO-Crate can support this process by describing the dataset and its computational context, allowing the Broker to automatically interpret information about required environment configuration and processing steps. Access restrictions expressed in the access template can also influence how the environment is configured. For example, if an access condition stipulates that row-level data may not be inspected, the Broker can request a restricted TRE configuration that prevents direct data inspection (e.g., similar to SURF’s Blind SANE environments). If multiple datasets from different holders are approved for linkage, the Broker can trigger a workflow in which datasets are staged in a shared workspace, potentially after linkage has been performed by a trusted third party where required by policy. Many of these steps normally involve extensive manual coordination between researchers, data providers, and infrastructure operators; the Broker automates and standardises these workflows, executing the permissions granted by the access template in a controlled manner.
- **Audit and Logging:** Every submitted access request, data holder decision, and Broker action is recorded, creating a comprehensive audit trail and supporting the data holder’s bookkeeping obligations. This audit trail documents the full lifecycle of a request, for example recording that “Researcher X accessed dataset Y under access condition Z on [date], performed analysis in Environment S, and exported output on [date] after approval by the data holder.” Such records support compliance and access provenance, allowing data holders to demonstrate accountability (e.g., under GDPR) and enabling funders or ethics boards to verify that data is used in accordance with the approved conditions. Because access conditions are expressed in standardised, machine-readable access templates, the resulting audit logs can also be structured and standardised. This facilitates aggregation and reporting across datasets and requests – for example, analysing how often certain access conditions are used, the average time required for approval, or how frequently requests require manual intervention – thereby providing insights that can help data holders refine their access policies over time.
- **Transparency**: The Broker provides a clear, standardised overview of how each access request is evaluated against the applicable ODRL-based access conditions. By explicitly indicating which obligations and constraints were satisfied or not satisfied, and which conditions contributed to approval or rejection, the Broker introduces a level of transparency that is largely absent in current data access procedures. In many current workflows, access decisions are made through opaque or ad-hoc processes, leaving applicants with limited insight into the reasoning behind approvals or denials – a challenge also documented in analyses of data access governance [17]. A condition-driven evaluation therefore improves the clarity and perceived fairness of access workflows by making the basis of decisions visible and consistent. In addition, the Broker’s audit logs and bookkeeping records provide a provenance trail of the full access process, further strengthening trust, accountability, and compliance for both data holders and data consumers.
- **User Support and Communication:** The Broker also manages notifications and facilitates communication between data consumers and data holders throughout the access workflow. If a request requires additional information or adjustments to satisfy a condition, the Broker can notify the data consumer and indicate the required action (for example, “Your project is approved, but you must complete the institute’s data security training before data can be accessed – please complete this step.”). The Broker also informs users when datasets have been successfully staged in a TRE or when other workflow milestones are reached. In cases where manual review is required, such as for particularly sensitive datasets, the Broker provides data holders with a dashboard to review and approve requests efficiently. This interface presents the request alongside the evaluation of the applicable access template, clearly showing which conditions have already been verified automatically. As a result, human reviewers can focus on the remaining judgement calls rather than repeating routine checks, significantly reducing administrative burden.

In effect, the Data Access Broker operationalises the standardised access conditions. It ensures that the intent of the ODRL rules is actually carried out in practice, without placing that burden on either the data consumer or the data holder each time. For the data consumer, the Broker makes the process feel simpler – a one-stop submission, then the data appears in a secure “research room” when ready. This addresses the trust issue: holders remain confident because the enforcement is automated and reliable (indeed, *“data holders remain in full control of their data”* when such mechanisms are in place).

Another advantage is in multi-party scenarios: if a data consumer requests data from data holder A and B and wants to combine them, the Broker can handle the joint conditions. Suppose holder A’s access condition says “allowed in SURF secure environment” and B’s says “allowed in SURF secure environment with data holder output check”. The Broker sees that both require SURF SANE, so it creates one SANE instance for the data consumer that both holders can access for oversight. It would enforce the stricter output check, ensuring both holder A and B approve the output. This can reduce the need for data consumers to manage multiple environments or parallel procedures when datasets from different holders share compatible access conditions. In situations where combining datasets triggers stricter safeguards or requires a jointly defined access condition, the Broker can apply that harmonised condition and configure a single secure environment accordingly. This ensures that the most stringent requirements are respected, while still avoiding unnecessary duplication of workflows for the data consumer.

Finally, the Broker concept is scalable. Once set up for the Netherlands, it could interoperate with brokers in other countries. It also could be extended to not just academic data consumers but other legitimate users (condition analysts, etc.) with proper credentialing.

## Future Outlook

While the core focus of this paper is on *access conditions* – the obligations, prohibitions, and requirements that must be met before access is granted – many data holders also attach use conditions that apply *after* access has been provided which are typically outlined in a licence. These conditions govern how the data consumer may work with the data, what safeguards must remain in place, and which responsibilities continue throughout the duration of the project. Examples include not attempting re-identification, not redistributing data, destroying or returning data after the project, and acknowledging the data source. Today, these types of use conditions are typically enforced through a mixture of trust, manual oversight, and policy documents, with substantial variation across organisations. A future evolution of the Data Access Broker could help bring structure, transparency, and efficiency to this process.

Another longer-term ambition of this initiative is to bring full alignment across the different policy instruments that govern sensitive data: licences, access conditions, use conditions, and the contracts that data consumers must accept. Today, these artefacts often originate from different sources – legal teams, repositories, TRE operators, institutional templates – and are expressed in different formats that are not always internally consistent.

### From Access Management to use Management

Once the Broker is already mediating the access phase, it becomes well positioned to support, though not fully enforce, selected aspects of responsible data use. Building on the foundation of machine-actionable access conditions, a next step would be the ability to process and monitor machine-actionable use conditions. In practice, this could include:

- **Monitoring ongoing obligations (light-weightcompliance support):** The Broker could keep track of project timelines, send reminders when obligations come due (e.g., end-of-project data destruction), or notify data holders when a dataset is nearing its access expiry date.
- **Technical support for selected use conditions:** Some use conditions can be supported after access has been granted, but only where this does not overlap with the environment-level safeguards that are already enforced during access (as described in the workflow automation section). Possible examples include automatic application of time-based restrictions such as closing access when the permitted period expires, and checking whether the data holder was acknowledged in publications based on the data access. These forms of support do not replace organisational responsibility, but they can help holders ensure that post-access requirements are handled consistently and transparently.
- **Providing a transparent record of use**: By logging actions already performed on behalf of the data holder, such as output vetting events, windowed access periods, or environment changes, the Broker can help create a standardised audit trail for data use, completing the data access audit trail. This reduces administrative burden and supports regulators or ethics committees in verifying appropriate data use.

Ultimately, incorporating use conditions into a machine-actionable framework would allow the Data Access Broker to support the *full lifecycle* of controlled data access:

- **Before access** (access conditions for automated matching and approval).
- **During access** (use conditions for monitoring, reminders, environment-level controls).
- **After access** (closing access, confirming obligations, recording compliance).

This vision aligns with broader European developments around digital rights vocabularies and execution frameworks, and it positions the Netherlands to lead in transparent, automated, and sustainable sensitive data governance.

### Toward a Fully Aligned Policy Stack

In a future phase, different artefacts that govern sensitive data could all be represented in a single ODRL-based policy framework, using one shared vocabulary for permissions, prohibitions, obligations, and constraints. This would include not only access conditions and use conditions, but also formal licences and contracts data consumers often sign, ensuring that human-readable legal text and machine-readable enforcement logic always remain matched.

In this model, the Data Access Broker becomes not only an access coordinator but also a policy harmonisation engine, ensuring that what the licence allows, what the access condition requires, what the use condition obliges, and what the user signs are all semantically aligned.

Such alignment reduces contradictions that frequently occur today – for example, when a repository’s licence permits one action while a data holder’s contract restricts it, or when “soft” obligations in policy documents are not reflected in system-level controls. By grounding all artefacts in the same machine-actionable framework, we can create predictable, transparent, and fully traceable data governance across the entire lifecycle of sensitive data access.

### Generalisability Beyond Social Sciences

Although this work focuses on administrative and social science data, the proposed architecture is not inherently tied to a particular research domain. We deliberately chose this domain because it combines a clear need for interoperable access procedures with an active community already working towards harmonisation across repositories, Trusted Research Environments, and national research infrastructures. This provides an appropriate setting in which to demonstrate the practical value of machine-actionable access conditions.

At the same time, the underlying design is intentionally modular. The Data Access Broker, ODRL policy representation, and reusable access templates are conceived as domain-independent building blocks, while domain-specific legal requirements, governance models, and vocabulary extensions can be incorporated as separate policy profiles. This allows the common orchestration and interoperability mechanisms to remain unchanged while accommodating differences between scientific disciplines.

We do not expect all research domains to converge immediately on a single access model. Areas such as health, genomics, defence, or commercial research often operate under additional legislative requirements and more fragmented governance structures. Rather than attempting to solve these differences from the outset, we envision the administrative and social science domain as a reference implementation that demonstrates the operational benefits of machine-actionable access conditions. As experience is gained, additional domain-specific profiles and policy vocabularies can be incorporated while preserving a shared interoperability layer across domains.

## Data Availability

No new data were created or analysed in this study. The paper presents a conceptual framework and does not report on empirical research involving datasets. All referenced publications and standards are publicly available and cited accordingly.

## Footnotes

1
www.w3.org/TR/odrl-model

2
odissei-data.nl

3
portal.odissei.nl

4
wetten.overheid.nl/BWBR0015926/2025-09-01

5
surf.nl/en/themes/research-infrastructure/sane-secure-environment-for-analysing-sensitive-data

6
https://www.surf.nl/en/themes/data-management-processing/sensitive-data-management-in-practice

## Abbreviations

AVG:	Algemene Verordening Gegevensbescherming (Dutch implementation of the GDPR)
BSN:	Burgerservicenummer (Dutch citizen service number)
CLARIAH	Common Lab Research Infrastructure for the Arts and Humanities
DANS	Data Archiving and Networked Services
DAB	Data Access Broker
EHDS	European Health Data Space
EOSC	European Open Science Cloud
FAIR	Findable, Accessible, Interoperable, Reusable
GDPR	General Data Protection Regulation
HDH	Health Data Hub (France)
HDAB	Health Data Access Body
LISS	Longitudinal Internet Studies for the Social Sciences
ODISSEI	Open Data Infrastructure for Social Science and Economic Innovations
ODRL	Open Digital Rights Language
ONS	Office for National Statistics (UK)
RO-Crate	Research Object Crate
RIVM	Rijksinstituut voor Volksgezondheid en Milieu (Dutch National Institute for Public Health and the Environment)
SANE	Secure ANalysis Environment
SKOS	Simple Knowledge Organisation System
SSH	Social Sciences and Humanities
TRE	Trusted Research Environment
W3C	World Wide Web Consortium
